# ReCLIP (Reversible Cross-Link Immuno-Precipitation): An Efficient Method for Interrogation of Labile Protein Complexes

**DOI:** 10.1371/journal.pone.0016206

**Published:** 2011-01-20

**Authors:** Andrew L. Smith, David B. Friedman, Huapeng Yu, Robert H. Carnahan, Albert B. Reynolds

**Affiliations:** 1 Department of Cancer Biology, Vanderbilt University, Nashville, Tennessee, United States of America; 2 Mass Spectrometry Research Center, Department of Biochemistry, Vanderbilt University, Nashville, Tennessee, United States of America; University of Hong Kong, Hong Kong

## Abstract

The difficulty of maintaining intact protein complexes while minimizing non-specific background remains a significant limitation in proteomic studies. Labile interactions, such as the interaction between p120-catenin and the E-cadherin complex, are particularly challenging. Using the cadherin complex as a model-system, we have developed a procedure for efficient recovery of otherwise labile protein-protein interactions. We have named the procedure “ReCLIP” (Reversible Cross-Link Immuno-Precipitation) to reflect the primary elements of the method. Using cell-permeable, thiol-cleavable crosslinkers, normally labile interactions (i.e. p120 and E-cadherin) are stabilized in situ prior to isolation. After immunoprecipitation, crosslinked binding partners are selectively released and all other components of the procedure (i.e. beads, antibody, and p120 itself) are discarded. The end result is extremely efficient recovery with exceptionally low background. ReCLIP therefore appears to provide an excellent alternative to currently available affinity-purification approaches, particularly for studies of labile complexes.

## Introduction

Identifying functionally relevant protein-protein interactions remains a significant problem in discovery-based research. Affinity purification coupled with Mass Spectrometry (MS) analysis is a rapid, sensitive, and unbiased method for identifying novel protein-protein interactions. While ongoing technical advances have dramatically improved the sensitivity and efficiency of mass spectrometry instruments and methods, most experiments are limited by the quality of the sample itself. Current methods represent a compromise where recovery is sacrificed for specificity or vise versa. Conventional co-immunoprecipitation by itself is invariably accompanied by unacceptable background. A common solution is to add a second affinity purification step. This Tandem-Affinity-Purification (TAP-tag) approach, however, minimizes background at the expense of transient and/or weak interactions that are lost because of the additional processing [1], [2].

Here, we have used p120-catenin (hereafter p120) and the E-cadherin complex as a model to develop an approach that captures labile interactions without sacrificing specificity. p120-catenin was originally identified as a prominent Src substrate, and later as a core component of classical cadherin complexes [3], [4]. Cadherins constitute a family of trans-membrane homophilic cell-cell adhesion receptors that interact in trans to link adjacent cells [5]. Adhesiveness is regulated, in part, by the catenins (i.e., α-catenin, β-catenin, γ-catenin/plakoglobin and p120-catenin) which bind to the cytoplasmic tail to form the cadherin complex [6]. β-catenin and γ-catenin directly bind to cadherins in a mutually exclusive manner, and physically and/or functionally link the complex to α-catenin and the actin cytoskeleton [7], [8].

Whereas β- and γ-catenins bind cadherins with high affinity under a variety of conditions, the p120 interaction is relatively labile. In RIPA buffer, for example, p120 is almost undetectable in cadherin immunoprecipitates, whereas the other catenins are efficiently recovered. Gentler detergents (i.e. NP-40) improve recovery, but are nonetheless relatively inefficient [4]. Digitonin can effectively preserve p120 binding in some cell types, but appears to act selectively on soluble (as opposed to cytoskeleton tethered) complexes [4], [9] and previous attempts using TAP methods have been unsuccessful due to extremely low recovery of p120 complexes (unpublished observations).

Chemical crosslinkers have been employed to stabilize protein-protein interactions for structural studies [10], or to demonstrate interaction between already suspected binding partners [11]. For example, it has been used successfully to capture transient dimerization of the Epidermal Growth Factor Receptor in response to ligand [12]. In particular, the cell-permeable, lysine-reactive crosslinker Dithiobis[succinimidyl propionate] (DSP, also called Lamont's Reagent) has been successfully used to facilitate co-immunoprecipitation of weakly interacting binding partners [13]. Recently, DSP-crosslinking has been combined with affinity-purification and mass spectrometry to identify novel binding partners [14], [15], suggesting that in-cell crosslinking can be used to characterize weak and transient complexes by mass spectrometry.

Here, using p120 and the cadherin complex as a model system, we describe an efficient approach that employs cell-permeable, thiol-cleavable crosslinkers to stabilize normally labile interactions (i.e. the p120 - E-cadherin interaction) *in vivo* prior to cell lysis and affinity purification. In our model, p120 was directly immunoprecipitated under stringent conditions and binding partners were selectively eluted from the p120 “bait” by chemical cleavage of the crosslinker. Unlike other approaches, this elution scheme removes the target protein along with the beads and antibody from the final sample, resulting in very low background. Western blot and MS analyses revealed that all core components of the cadherin complex were efficiently recovered along with several novel candidates for direct or indirect p120 binding partners. This approach, which we have termed ReCLIP (Reversible Cross-Link Immuno-Precipitation) is simple and produced remarkably clean preparations of p120 binding partners for proteomic analyses. These results suggest that ReCLIP provides high sensitivity without sacrificing specificity, and therefore provides a robust alternative to other affinity-purification methods.

## Results

### Determination of optimal crosslinker concentrations

**Figure 1:**
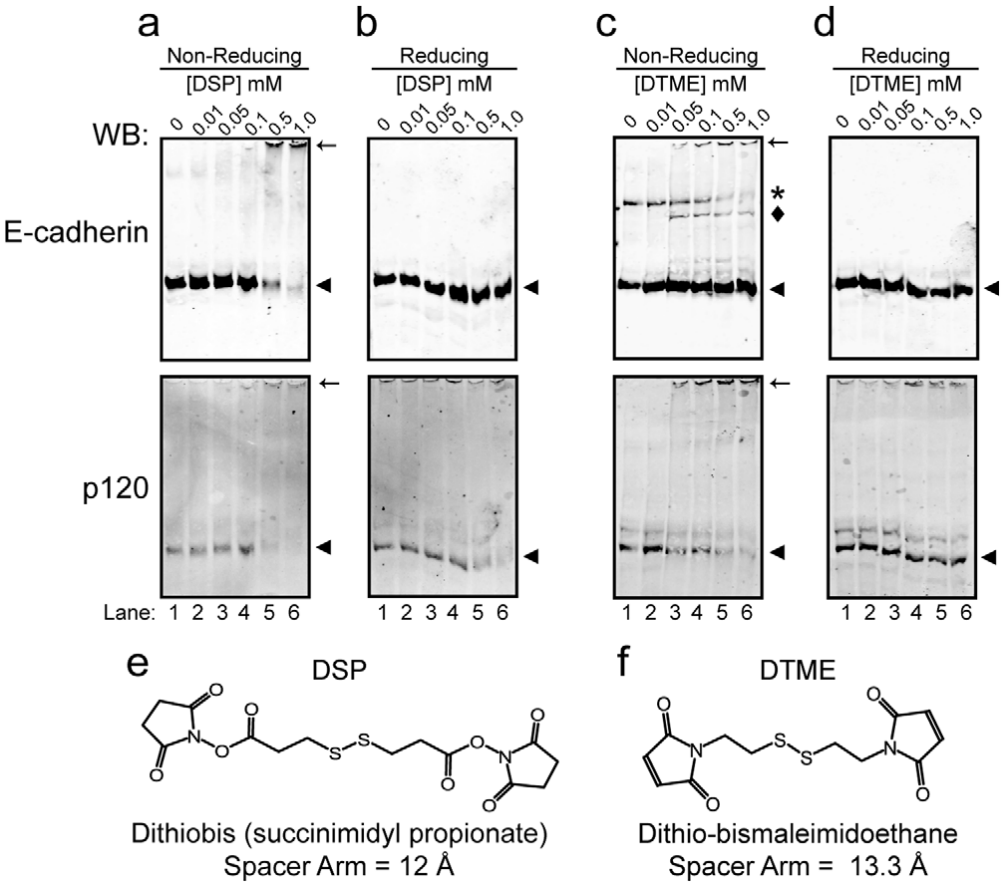
Titration of intracellular cross-linking of p120 and E-cadherin. Western blot analysis of E-cadherin (top panels) and p120 (bottom panels) in whole cell lysates of A431 cells treated with the indicated concentrations of DSP (a, b) or DTME (c, d) between 0.01 mM and 1.0 mM. Samples were prepared under non-reducing (a, c) and reducing conditions (b, d) as indicated. Arrowheads indicate monomeric E-cadherin and p120, large cross-linked species are indicated with arrows, smaller crosslinked E-cadherin species are indicated with a diamond (⧫), and a possible cysteine-induced E-cadherin dimer is indicated with an asterisk (*). The chemical structures of DSP (e) and DTME (f) are shown, images were constructed with the DrawIt application in KnowItAll Informatics System v. 4.1 (Bio-Rad).

We initially identified candidate crosslinkers and evaluated conditions for use. Two specific crosslinkers, Dithiobis[succinimidyl propionate] (DSP) and Dithio-bismaleimidoethane (DTME), were chosen based on their distinct chemical properties. DSP reacts with primary amines and has a spacer-arm of 12 Å (Figure 1e), forming crosslinks between lysine residues of interacting proteins. DSP has been commonly used in a variety protein-interaction studies [16], [10] due in part to the high abundance of lysine residues in proteins. DTME reacts with sulfhydryl groups and has a spacer arm of 13.3 Å (Figure 1f), forming crosslinks between cysteine residues of interacting proteins. DTME would be expected to produce fewer crosslinks, however it may capture interactions that DSP cannot. While not commonly used, DTME has been successfully applied to protein-protein interactions studies [17]. Importantly, both compounds are cell-permeable, allowing for in-cell crosslinking of endogenous complexes prior to cell-lysis. Additionally, both compounds are thiol-cleavablez, allowing for “reversal” of the crosslinks via chemical cleavage by a reducing agent (i.e. DTT).

Optimal crosslinker conditions were determined using A431 epidermoid carcinoma cells, a human epithelial cell line that has been used for a number of cell-cell adhesion studies [18], [19]. A431 cells were washed with PBS and exposed for 30 minutes to increasing concentrations of DSP or DTME (Figure 1) in PBS, pH 7.4. Cells were then lysed at 4°C in RIPA and the lysates treated for 15 min with DTT (reducing, panels b and d) or not (nonreducing, panels a and c), as indicated. Samples were then analyzed by SDS-PAGE, followed by Western blotting for E-cadherin (top panels) or p120 (bottom panels).

Figure 1a shows a dose-dependent reduction in monomeric E-cadherin (top panel, arrowhead) and the simultaneous appearance of crosslinked complexes across the top of the gel that are too large to resolve (arrow). Note that the monomeric E-cadherin (and p120, lower panel) is decreased at 0.5 mM DSP and almost absent at 1.0 mM, indicating that the vast majority of E-cadherin and p120 is crosslinked into high molecular complexes at these concentrations. Figure 1b shows that monomeric protein is efficiently recovered by addition of DTT. Note that at 0.5 mM DSP, virtually all of the monomeric E-cadherin and over half of the monomeric p120 are recovered (compare lanes 6 in a and b, upper and lower panels respectively), and that the high molecular weight bands are no longer present. It is not entirely clear why the recovery of p120 in whole cell lysates is less efficient than that for E-cadherin. The difference, however, is not generally observed in immunoprecipitates, suggesting that the phenomenon may reflect competition for reducing agent among the large number of crosslinked proteins present in the whole cell lysate.

Crosslinking with DTME was less efficient, as evidenced by the relatively high levels of monomeric E-cadherin remaining at the 1.0 mM dose (panel c, compare lanes 1 and 6). This is consistent with the lower abundance of cysteine residues relative to lysine. Nonetheless, the appearance of progressively larger E-cadherin-containing complexes with increasing DTME indicates the presence of crosslinked species. The faster migrating band (Fig. 1c, diamond) probably represents a partial complex. The exact content is not known, but p120 is clearly absent. Further crosslinking generates p120-containing higher order complexes, which are too large to resolve by SDS-PAGE (arrow). In these non-reduced samples, an additional E-cadherin band is present even in the absence of cross-linker (asterisk). The precise identity of this E-cadherin complex is unclear, but it may represent cadherin dimers caused by the addition of cysteine to quench the DTME crosslinking reaction, as dimerization is induced, in part, by cysteine mediated disulfide bonds within the extracellular domain [20], [21]. Interestingly, for reasons not entirely clear, DTME appears to crosslink p120 more efficiently than E-cadherin, as evidenced by significant loss of monomeric p120 (panel c, compare lanes 1 through 6).

Based on these data, we chose 0.5 mM DSP and 0.5 mM DTME as optimal concentrations for subsequent experiments. In the case of DSP, 1.0 mM was more effective than 0.5 mM, but we chose the lesser of the two to limit nonspecific capture. For DTME, there was no apparent difference between 1.0 and 0.5 mM so the lesser amount was used.

### Efficacy, efficiency, and specificity of crosslinking with DSP and DTME

**Figure 2:**
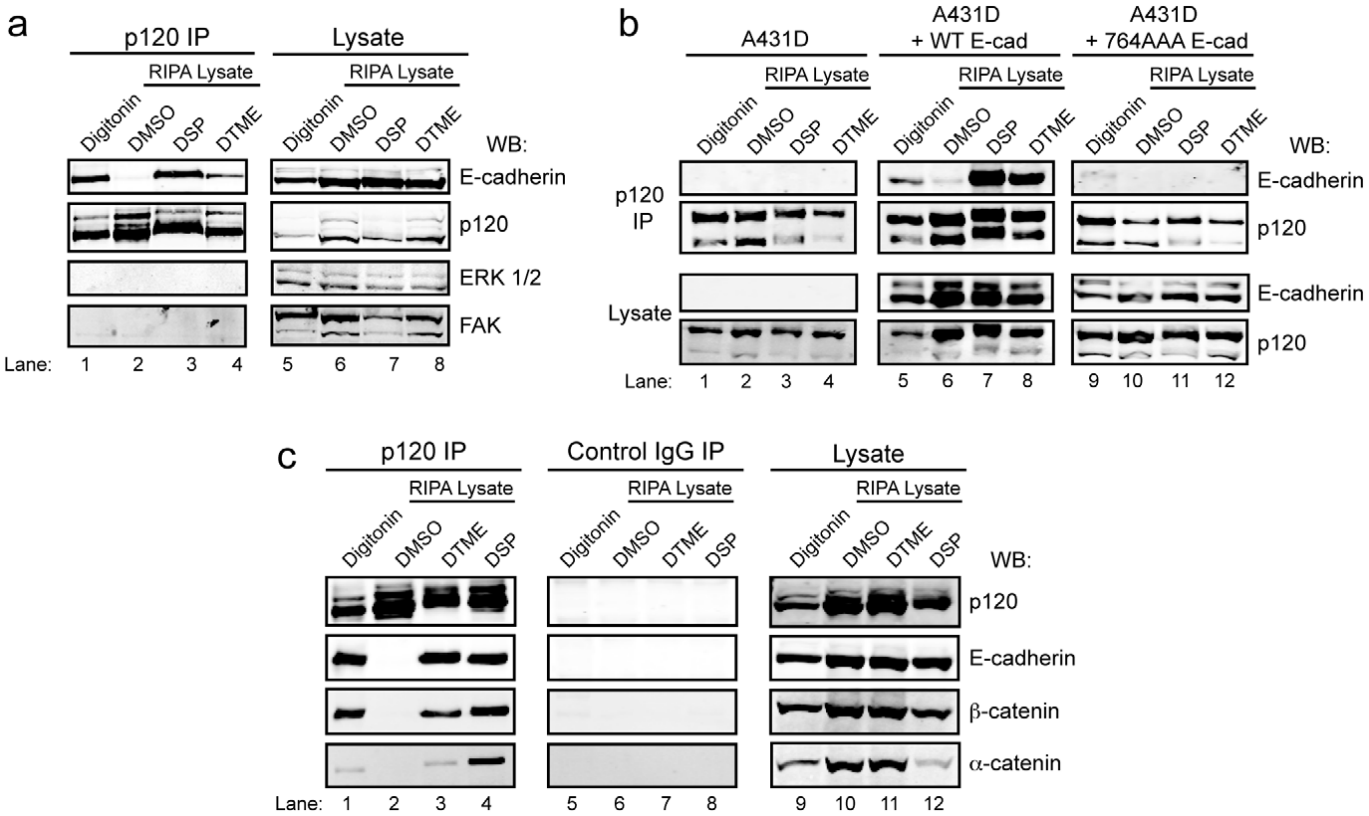
In-cell cross-linking preserves the interaction of p120 and E-cadherin and is specific for interacting proteins. (a) Western blot analysis of p120, E-cadherin, FAK, and p42/44 MAPK in p120 immunoprecipitates and lysates from A431 cells lysed in 1% digitonin or RIPA buffer following treatment with DMSO vehicle or 0.5 mM cross-linker as indicated. (b) Cadherin-negative A431-D cells, and A431-D cells stably expressing wild type (WT E-cad) or p120-uncoupled (764 E-cad) E-cadherin were prepared and analyzed as in A. (c) Western blot analysis of p120, E-cadherin, β-catenin, and α-catenin in p120, control IgG immunoprecipitates, and lysates from A431 cells treated as in panel a.

Next, we used the E-cadherin – p120 interaction as a model to assess the efficacy of DSP and DTME under the above conditions. The amount of E-cadherin co-immunoprecipitating with p120 was determined after in-cell crosslinking with DSP or DTME (Figure 2a). Cell lysis in a digitonin-containing buffer (without crosslinking) was used as a reference (Figure 2a, lane 1), because it is relatively effective in A431 cells at preserving the p120 – E-cadherin interaction [9]. In contrast, the remaining samples were treated with DSP, DTME, or vehicle alone (DMSO), as above, and lysed in RIPA buffer. p120 was then immunoprecipitated from all samples, eluted in reducing LSB, and analyzed by SDS-PAGE and Western blotting.

Figure 2a shows that E-cadherin recovery from p120 immunoprecipitates after DSP crosslinking was as good, if not better, than that obtained from the digitonin lysate (compare lanes 1 and 3). DTME was less efficient (lane 4), whereas no E-cadherin was recovered in the absence of crosslinker (i.e. DMSO, lane 2). Thus, E-cadherin was not recovered in RIPA alone, but crosslinking with DSP preserved the interaction. Moreover, irrelevant cytoplasmic (i.e. MAP Kinase) and membrane-associated (i.e. Focal Adhesion Kinase) proteins were absent from the p120 immunoprecipitates but clearly present in whole cell lysates. Thus, DSP and DTME crosslinking appears to be quite specific under these conditions.

To further assess specificity, we asked whether E-cadherin and p120 could be crosslinked under conditions where physical interaction is selectively uncoupled (Figure 2b). We previously described a minimal E-cadherin mutant (E-cad 764AAA) that is physically uncoupled from p120 but nonetheless forms cell-cell junctions and interacts normally with β-catenin [22]. In Figure 2b, we introduced WT (lanes 5–8) or mutant (lanes 9–12) E-cadherin into the A431D cell line, a cadherin-negative A431 variant. The absence of E-cad 764AAA in p120 immunoprecipitations (lanes 9–12, top panel) shows clearly that this mutant is not crosslinked to p120, implying that direct physical interaction is indeed essential. In contrast, WT E-cadherin is efficiently crosslinked (lanes 5–8, top panel).

To further test the efficacy of crosslinking, we extended the analysis to α- and β-catenins, which form a tertiary (indirect) complex with p120 via E-cadherin (Figure 2c). Interestingly, the entire complex is efficiently crosslinked by DSP (lane 4). α-catenin, in particular, was easily recovered relative to the DTME or digitonin methods. The middle panels (lanes 5–8) show that negative-control immunoprecipitation with a p120 monoclonal antibody that does not recognize human p120 (control IgG, mAb 8D11) under conditions identical to the first panel (lanes 1–4) does not bring down members of the cadherin complex.

### Reversible Cross-Linking Immuno-Precipitation (ReCLIP) for Mass Spectrometry

**Figure 3:**
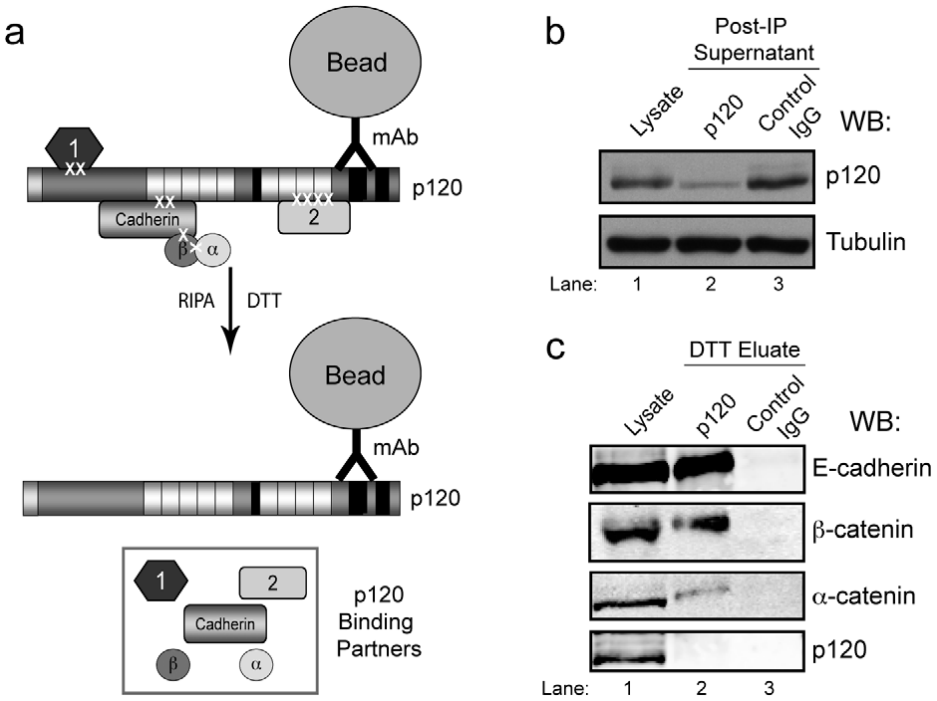
Elution of binding partners from p120. (a) A schematic of the elution strategy. Following immunoprecipitation and washing of cross-linked complexes on p120 mAb beads, binding partners are released by incubation with DTT in RIPA buffer, cleaving the cross-links and releasing interacting proteins from p120. (b) A representative western blot demonstrating depletion of p120 from A431 cell lysates following immunoprecipitation with p120 mAb beads of control IgG beads. Tubulin is shown as a loading control. (c) Elution of known binding partners, but not p120, from p120 mAb beads. Whole cell lysate is shown as a control, and 10% of the DTT eluate was analyzed for E-cadherin, β-catenin, α-catenin, and p120 by Western blot.

Figure 3 illustrates the procedure we have developed for rapid and clean isolation of binding partners for MS analysis. The schematic (panel 3a) shows immunoprecipitation of a crosslinked p120 complex followed by selective elution of the individual components. Binding partners are efficiently recovered by breaking the crosslinks with reducing agent, essentially reversing the procedure. With the antibody covalently bound to the bead (see bead preparation under Materials and Methods), DTT releases crosslinked binding partners only. The most abundant protein ‘contaminants’, mAb 15D2 and mAb-bound p120 itself (the bait), are discarded along with the beads, resulting in a highly purified mixture of eluted binding partners. Panel b illustrates the efficiency of the immunoprecipitation, as evidenced by depletion of p120 from the supernatant (panel b, compare lanes 1 and 2). Panel c shows that the coimmunoprecipitated E-cadherin is efficiently recovered by DTT elution (panel c, top panel, compare lanes 1 and 2) while p120 is essentially absent, having been discarded with the beads (panel c, bottom panel, lane 2). Furthermore, immunoprecipitation using control IgG (mAb 8D11) does not deplete p120 from the lysate (panel b, lane 3) or and E-cadherin and associated catenins are not detected in the DTT eluate (panel c, lane 3).

### Efficacy of ReCLIP

To test the efficacy of ReCLIP, p120 and control elutions from A431 cells crosslinked with DSP were subjected to shotgun analysis by single-dimension liquid chromatography tandem mass spectrometry (LC-MS/MS). Core p120 binding partners were easily identified, as evidenced by high spectral counts for E-cadherin and the catenins (Table 1). Note, however, that spectral counts are only partly indicative of protein abundance. For example, E-cadherin is consistently under-represented relative to its size, which is similar to the catenins. Importantly, cadherin proteins are not detected control pull downs (i.e. zero peptides), as shown in Table 1. Nonspecific background (ie, proteins detected in both experimental and control samples) was remarkably low, consisting primarily of common artifacts such as chaperones, metabolic proteins, and highly abundant cytoskeletal proteins, as illustrated in Table S1.

**Table 1:** Recovery and identification of core p120 binding partners using ReCLIP.

*Protein*	*UniProt* *Accession*	*Average spectral countin p120 IP*	*Standard* *Error*	*Average spectral countin control IP*
E-cadherin	IPI00000513.1	9	2.11	0
α-catenin	IPI00215948.4	38	9.17	0
β-catenin	IPI00017292.1	24	6.07	0
Plakoglobin	IPI00554711.2	12	5.08	0

Average spectral totals for E-cadherin, α-catenin, β-catenin, and Plakoglobin from 3 independent ReCLIP experiments from A431 cells treated with DSP. No peptides for these proteins were identified in the corresponding control samples.

### Effects of simultaneous DSP and DTME crosslinking

**Figure 4:**
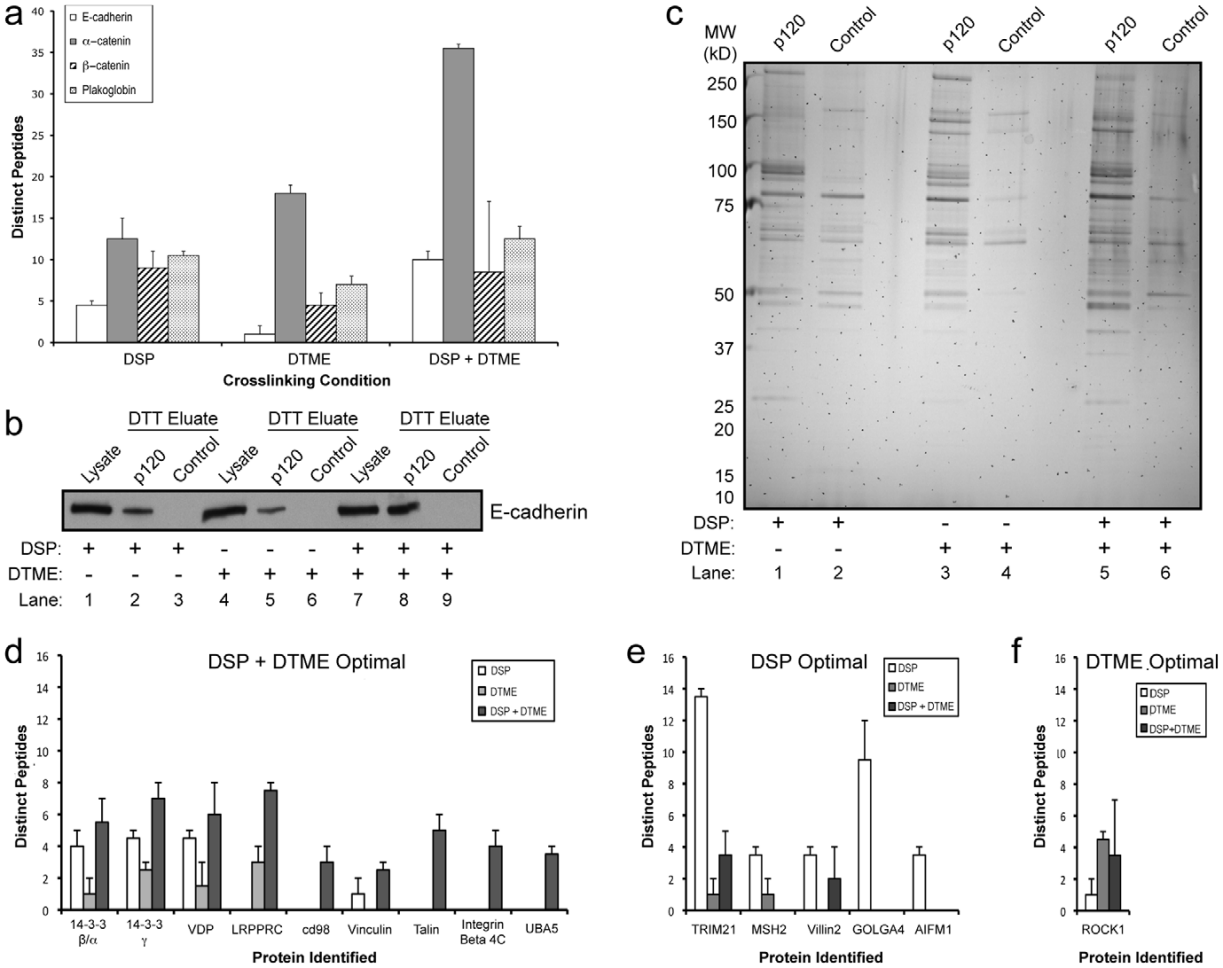
Cross-linkers can be combined to enhance complex recovery. (a) Average number of distinct peptides identified in 2 LC-MS/MS runs for E-cadherin, α-catenin, β-catenin, and plakoglobin from A431 cells treated with DSP, DTME, or both compounds simultaneously (DSP + DTME). Error bars represent standard error of the mean. Background levels were similar across all conditions. (b) Western blot analysis of E-cadherin levels in lysates (Lysate), p120 eluates (p120), and control IgG eluates (Control) from A431 cells treated with the indicated cross-linkers. (c) Silver stain analysis of total protein recovery from p120 and control IgG eluates from each condition (DSP, DTME, or DSP + DTME). (d–f) Average distinct peptide recovery of 15 additional putative p120 binding partners under each cross-linking condition. Proteins were grouped based on whether more peptides were detected using the combination of DSP and DTME (d), DSP alone (e) or DTME alone (f).

zNext, we asked whether use of DSP and DTME together is more efficient than either one alone. Figure 4a shows the number of distinct peptides (per protein) of cadherin complex proteins detected using individual or combined crosslinkers. Complete peptide identification data (including peptide sequences and cross-correlation scores) is provided in Table S2. For E-cadherin and α-catenin, combining DSP and DTME was clearly more efficient than individual usage, whereas no little or no improvement was observed for β-catenin and Plakoglobin. The same result is illustrated by Western blotting (Figure 4b) using E-cadherin as the readout. In the experiment shown, the DSP + DTME combination was highly effective (compare lanes 7 and 8), whereas each compound by itself was less efficient (compare lane 1 and 2, and lane 4 and 5).

The efficacy of ReCLIP under three crosslinking conditions was further evaluated by SDS-PAGE and silver staining (figure 4c). The data indicate an excellent signal to noise ratio across all three conditions, with very few bands detectable in control IgG lanes (lanes 2, 4, and 6). For each condition, some of the bands were unique, as expected. Notably, combining DSP and DTME captured most of the individual bands observed with either crosslinker alone while background remained remarkably low (compare lanes 5 and 6). These data indicate that regardless of the crosslinker used, ReCLIP provides robust recovery with very low background.

In addition to the core components of the cadherin complex, we identified at least 15 unique candidate p120 binding partners in MS analysis, and grouped them according to the condition that resulted in the highest number of peptide hits (Figure 4d–f). For example, Figure 4d contains the candidates for which DSP and DTME together yielded more hits than DSP (4e) or DTME (4f) alone. The cutoff for inclusion was a minimum of two hits against a background of zero, although the majority exceeded these criteria. Complete peptide identification data for these candidates is provided in Table S2. As expected, the highest number of peptide hits for most of the candidates was obtained when DSP and DTME were combined (panel d). However, for five of the candidates, the highest number of hits was obtained using DSP alone (Figure 4e), whereas DTME was optimal for only one protein (Figure 4f). Interestingly, five of the candidates were captured only when DSP and DTME were used together. On the other hand, combining DSP and DTME prevented capture of three candidates (MSH2, GOLGA4, and AIFM1). In general, the use of both DSP and DTME together was most effective in that the majority of candidates (12/15) were detected and only three were missed. With DSP or DTME alone, just over half of the candidates (8/15) were missed. Overall, these data suggest that the most effective approach is to combine DSP and DTME, but this approach may not be ideal for all proteins. Thus, it is recommended that investigators test each crosslinker individually and in combination in order to determine the appropriate ReCLIP condition for a given target protein.

## Discussion

Here, we have used reversible in-cell crosslinking to develop an extremely efficient method (ReCLIP) for studying protein complexes by mass spectrometry. The component techniques by themselves are not necessarily novel, but they are uniquely combined and optimized to generate a powerful method for studying labile complexes. The single immunoprecipitation approach minimizes sample loss, a common problem in TAP methods. Furthermore, covalent crosslinking preserves relevant interactions despite stringent lysis and washing conditions that reduce background. Thus ReCLIP appears to be particularly powerful for studying labile protein interactions that in principle could be lost using TAP approaches.

Among the several optimized parameters of the ReCLIP method, two in particular turn out to be critical. First, in-cell crosslinking covalently stabilizes endogenous interactions (as they occur *in vivo*). Thus, weak or transient interactions are captured in situ and retained, regardless of subsequent lysis and washing conditions, until the very end of the procedure when the product is eluted. Second, the elution method itself is both gentle and highly selective. A major difference between ReCLIP and other methods is that only putative binding partners are eluted when the crosslinks are cleaved (see Figure 3a). Thus, beads, antibody, and other components of the solid phase, including the bait itself (in this case, p120) are completely absent from final sample. The removal of bait and immunoprecipitating antibodies from the sample is important because these are by far the most abundant protein contaminants present in most methods.

A potential consideration when using ReCLIP in conjunction with MS is that some of the recovered peptides are covalently bound by a cleaved crosslinker. After cleavage by reducing agent, half of each crosslinker remains attached to a target residue in the crosslinked protein. In addition, bound crosslinker may alter proteolytic cleavage patterns, as has been demonstrated for other lysine modifications [23]. In addition The cleaved crosslinker alters peptide mass and can prevent recognition by standard MS algorithms [24]. Both events (mass-shift and reduced cleavage) can reduce the number of peptides generated and/or detected. Such complications are not likely to affect the data significantly because crosslinked peptides represent only a small fraction of the total number generated following digestion of the sample with trypsin. The effect is further limited by using the minimal effective concentration of the crosslinker, as determined by preliminary titration experiments. It is also possible to identify modified peptides by re-analyzing the spectra using a subset database that allows for the extra mass (105.16 Da per Lysine for DSP and 159.21 Da per Cysteine for DTME) produced by the cleaved crosslinker [25].

One potential drawback to ReCLIP is that very low molecular weight proteins might be missed because there are fewer available sites for crosslinking, and fewer tryptic peptides to detect. For example, if a protein is crosslinked and contains only two tryptic peptides, one will be missed due to the crosslink modification. Such proteins would be overlooked because the score (one peptide against zero background) is below the cutoff for positive identification. Thus, small proteins (e.g. small GTPases such as RhoA) may be overlooked, because few unmodified peptides are available. Thus, it is important to consider protein size and peptide coverage when assessing proteins with relatively low peptide scores (e.g. two peptides against zero background).

ReCLIP has been optimized to study endogenous complexes using a monoclonal antibody. By design, this allows physiologically relevant complexes to be recovered with a relatively high degree of specificity. However, ReCLIP can still be used in conjunction epitope-tags (e.g., Flag, Myc, HA epitope tags) in cases where specific antibodies are not available. Protein overexpression, however, may increase nonspecific interactions. For example, we have noticed that components of the proteasome are selectively identified under such conditions. Presumably, the cell is targeting the excess protein for degradation and we are then crosslinking it to components of the proteasome. It is possible that we missed certain previously identified p120 binding partners (e.g., Kinesin Heavy Chain [26], [27]). for this reason, as the interaction between p120 and Kinesin Heavy Chain is more efficiently detected under conditions of p120 overexpression. Alternatively, the interaction may be different or absent in A431 cells.

Surprisingly, Kaiso was not identified as a p120 binding partner by ReCLIP. However, Kaiso is a relatively low abundance transcriptional repressor found primarily in the nucleus in cultured cells [28]. Thus, it is possible that spatial separation, low Kaiso expression, low interaction stoichiometry, or any combination thereof ultimately limits the sensitivity of ReCLIP. Of note, p120 and Kaiso can be detected by conventional co-immunoprecipitation in gentle detergent buffers [28], suggesting that low abundance of Kaiso is not by itself the limiting factor. Instead, cell lysis without prior crosslinking may actually facilitate such interactions by permitting the mixing of proteins from otherwise spatially separate pools (e.g. nuclear Kaiso and cytoplasmic p120). With ReCLIP, protein complexes are crosslinked in situ and then lysed in RIPA, a stringent buffer designed expressly to be compatible with antibody-antigen interactions while preventing nonspecific and/or weak interactions. Thus, some events that occur post-lysis (e.g. the p120-Kaiso interaction) will undoubtedly be prevented by the ReCLIP lysis and washing conditions. On the other hand, this feature of ReCLIP may allow one to selectively capture physiological complexes under a defined condition and time interval, potentially identifying interactions that occur transiently in response to a stimulus.

In addition to the cadherin complex, we identified several candidate p120 binding partners (Figure 4c–e and Table S2) including p160 Rho Kinase (ROCK1). ROCK1 is a prominent effector of RhoA that regulates the acto-myosin machinery and other signaling pathways [29]. This novel interaction, which will be described in a separate report (Smith *et al*. in preparation), is consistent with other known roles of p120. For example, p120 regulates the activity of RhoA [30] and can associate with p190 RhoGAP at the adherens junction [31]. ROCK1 has not been linked to p120 by other methods (e.g. conventional immunoprecipitation and TAP-Tag), consistent with the apparent increased sensitivity of ReCLIP. Interestingly, no Rho-family GTPases were detected using ReCLIP, including RhoA which has been reported to directly interact with p120 [32], [33]. A potential explanation for this result is the inherent bias of mass-spectrometry against small proteins. Nonetheless, the recovery of ROCK1 along with its substrate Villin-2/Ezrin suggests that a functional Rho complex associates with p120.

Another candidate binding partner, cd98 (also known as 4F2 Heavy Chain), appears to reflect capture of a tertiary interaction. In general, tertiary (as apposed to direct) interactions are considerably more difficult to capture by conventional methods, but in principle could be significantly stabilized by limited crosslinking. cd98 is an integral membrane protein that forms a heterodimer with the LAT-2 amino-acid transporter (also known as 4F2 Light Chain) [34]. cd98 also regulates β1-integrin clustering [35], [36], [37] and heterotypic cell-cell interactions [38]. Interestingly, a previous study suggested the recruitment of cd98 to cadherin-based cell-cell junctions [34]. Consistent with this report, we find that cd98 co-localizes precisely with E-cadherin and p120 in A431 cells (see Figure S1a). However, in E-cadherin reconstitution experiments (using the cadherin-negative A431 derivative, A431D) cd98 is also recruited to both wild type and p120 uncoupled E-cadherin complexes, indicating that the direct interaction is not with p120 itself, but instead to some other member of the E-cadherin complex (see Figure S1b). As with ROCK1, we have not detected cd98 by other methods. Importantly, the indirect association of p120 with cd98 provides additional evidence that ReCLIP can routinely capture tertiary interactions that would otherwise be lost, making it attractive for interactome mapping studies.

In summary, we have developed ReCLIP (Reversible Cross-Link Immuno-Precipitation), an approach designed expressly to retain weak interactions without sacrificing specificity and/or sensitivity. The procedure is relatively simple and yet generates excellent signal-to-noise ratios in MS analyses. Although we have focused on the cadherin complex as a model system, the method should be broadly applicable. Overall, ReCLIP offers a potentially powerful alternative to previously described affinity-purification approaches and appears to be particularly suitable for interrogating labile protein complexes.

## Materials and Methods

### Cell Lines and Cell Culture

A431 and A431D epidermoid cervical carcinoma cell lines were obtained from Dr. Margaret Wheelock (University of Nebraska Medical Center). A431D cells expressing wild type (WT) or 764AAA E-cadherin [22] were generated using the LZRS-MS-neo retroviral vector as described previously [39], [40]. All cells were cultured in DMEM (Gibco/Invitrogen) supplemented with 10% FBS (Hyclone) and 1% penicillin-streptomycin (Gibco/Invitrogen).

### In-cell chemical cross-linking

In-cell cross-linking was performed using Dithiobis[succinimidyl propionate] (DSP) and Dithio-bismaleimidoethane (DTME) (Pierce/Thermo Scientific). For each experiment, cross-linkers were freshly prepared as a 20 mM solution in Dimethyl Sulfoxide (DMSO) and diluted to the indicated final working concentrations in Phosphate-buffered saline, pH 7.4 (PBS, Fisher Scientific). Cells were washed twice with PBS at room temperature to remove all traces of media and incubated with the cross-linker solution for 30 minutes at room temperature. After removal of the cross-linker solution, cells were incubated at room temperature for 10 minutes with quenching solution (20 mM Tris-Cl pH 7.4, 5 mM L-Cysteine). Quenching solution was then removed and cell lysates were prepared as described below.

### Antibodies and Bead Preparation

The generation of monoclonal and polyclonal antibodies for p120 (pp120, 15D2, 8D11, F1αSH) has been described [41]. Of note, mAb 15D2 was used for all p120 immunoprecipitations, while mAb 8D11 is used as a control IgG because it does not recognize human p120. Other antibodies used include E-cadherin monoclonal antibody (BD Transduction), α-catenin rabbit polyclonal antibody (C-2081, Sigma), β-catenin rabbit polyclonal antibody (C-2206 Sigma), p42/44 MAPK rabbit polyclonal antibody (Cell Signaling), and Focal Adhesion Kinase (FAK) rabbit polyclonal antibody (C-20, Santa Cruz). Anti-cd98 monoclonal antibody 4F2 was a kind gift from Dr. Roy Zent [42], [43]. Secondary antibodies for western blot analysis include anti-mouse AlexaFluor 680 (Molecular Probes) and anti-rabbit IRdye 800 (Rockland Immunochemicals).

To prepare magnetic beads for immunoprecipitation, Protein G Dynabeads (Dynal/Invitrogen) were washed with Citrate Phosphate buffer pH 5.0 (25 mM citric acid, 50 mM dibasic sodium phosphate) and incubated with either 15D2 or 8D11 monoclonal antibodies for 2 hours at room temperature with end-over-end rotation. Bead-antibody complexes were washed with citrate phosphate buffer, followed by two washes with 0.2 M Triethanolamine (TEA) pH 8.2. Antibodies were covalently bound to Protein G beads by incubation 20 mM Dimethyl Pimelimidate (Sigma) in TEA for 30 minutes at room temperature with end-over-end rotation, followed by incubation for 15 minutes with 50 mM Tris to quench the crosslinking reaction. Subsequently, beads were washed three times with PBS-Tween. After washing with 0.1 M Glycine, pH 2.5 to remove non-covalently bound antibodies, beads where washed again with PBS-Tween and stored at 4°C.

### Conventional Immunoprecipitation and Western Blot Analysis

Lysis, immunoprecipitation, and western blot methods have been described previously [40]. Briefly, cells were lysed in Radioimmunoprecipitation Assay (RIPA) buffer (50 mM Tris pH 7.4, 150 mM NaCl, 1% NP-40, 0.5% deoxycholic acid, 0.1% SDS) or Digitonin buffer (20 mM Tris pH 7.5, 150 mM NaCl, 1% Digitonin) supplemented with protease and phosphatase inhibitors (1 mM PMSF, 5 µg/mL Leupeptin, 2 µg/mL Aprotinin, 1 mM EDTA, 50 mM NaF, and 1 mM NaVO_4_). Lysates were cleared by centrifugation and total protein concentrations were determined by BCA assay (Pierce/Thermo Scientific). For immunoprecipitation, the specified antibody was added to the clarified lysate for 2 hours at 4°C with end-over-end rotation, followed by incubation with Protein G sepharose (GE Healthcare) for an additional hour at 4°C. Beads were washed with lysis buffer, resuspended in 2× Laemmli Sample Buffer (LSB), and boiled for 5 minutes. Lysates were prepared in LSB or non-reducing sample buffer (50 mM Tris pH 6.8, 4% Glycerol, 1% SDS, 0.004% Bromophenol Blue) as indicated. Cross-linked lysates were incubated with 50 mM DTT for 15 minutes prior to boiling to ensure cleavage of disulfide bonds within the cross-linkers.

Immunoprecipitations and whole cell lysates were separated by SDS-PAGE and transferred to nitrocellulose membranes (Whatman) for western blotting. Non-specific binding to membranes was blocked with 3% nonfat milk in TBS (10 mM Tris pH 7.4, 150 mM NaCl), and membranes were incubated with primary antibody in milk overnight at 4°C. Membranes were incubated with secondary antibody in Odyssey blocking buffer (Li-Cor) for 1 hour at room temperature. Antibodies were detected using the Odyssey infrared imaging system (Li-Cor).

### Reversible Cross-Link Immunoprecipitation Procedure

Four 15 cm dishes of 90% confluent A431 cells (1×10^8^ cells) were used for each experiment. Cells were washed twice with freshly-prepared PBS pH 7.4 to remove all traces of media. Following removal of PBS, 10 mL of a 0.5 mM crosslinker solution in PBS (as described above) was added to the each plate. Cells were incubated with crosslinkers for 30 minutes at room temperature, with occasional agitation. Crosslinker solution was then removed, and 10 mL quenching solution was added to each plate for an additional 10 minutes. Following quenching, plates were placed on an ice bath and washed once more with chilled PBS, and lysed with freshly-prepared RIPA buffer plus protease and phosphatase inhibitors (1 mL RIPA buffer per dish). Lysates were homogenized using a 23-gauge needle and cleared by centrifugation. Equal volumes of clarified lysate were incubated with either 15D2 (p120) or 8D11 (control) bound Protein G Dynabeads for 3 hours at 4°C with end-over-end rotation. The beads were then washed 5 times with 1 mL RIPA buffer supplemented with protease and phosphatase inhibitors. p120 binding partners were eluted by incubating the beads with RIPA buffer supplemented with 50 mM DTT in for 30 minutes at 37°C with end-over-end rotation.

For mass spectrometry analysis, eluates were boiled in freshly prepared LSB, separated by SDS-PAGE on a Nu-PAGE 4–12% Bis-Tris gels (Novex/Invitrogen) and stained with “Blue Silver” colloidal coomassie stain [44]. The entire lane was excised and processed for shotgun analysis using single-dimension liquid-chromatography tandem-mass spectrometry (LC-MS/MS) by the Vanderbilt University Medical Center Proteomics Core. For silver stain analysis, 10% of the eluate was separated by SDS-PAGE and protein was visualized using Silver Stain Plus (Bio-Rad), according to the manufacturer's protocols. Following staining, gels were imaged using the FluorChem-8900 Gel Documentation System (Alpha Innotech).

### Mass Spectrometry and Protein Identification

Proteins were resolved by SDS-PAGE, and protein bands of interest were excised and cut into 1 mm cubes and equilibrated in 50 mM NH_4_HCO_3_. Proteins were then reduced within the gel pieces with DTT (3 mM in 100 mM NH_4_HCO_3_, 37°C for 15 min) followed by alkylation with iodoacetamide (6 mM in 50 mM NH_4_HCO_3_ for 15 min). The gel pieces were then dehydrated with acetonitrile and rehydrated with 15 µL 12.5 mM NH_4_HCO_3_ containing 0.01 µg/µL trypsin (Trypsin Gold, Promega), and trypsin digestion was carried out for >2 h at 37°C. Peptides were extracted with 60% acetonitrile, 0.1% formic acid, dried by vacuum centrifugation and reconstituted in 15 µL 0.1% formic acid. 5 µL of peptide hydrosylate were analyzed by C18 reverse-phase LC-MS/MS using a Thermo LTQ ion trap mass spectrometer equipped with a Thermo MicroAS autosampler and Thermo Surveyor HPLC pump, nanospray source, and Xcalibur 2.0 instrument control using standard triple-play methods. Tandem MS data were analyzed with the Sequest algorithm to search a human subset of the UniRef100 database (Jan 23 2007, 223514 entries) using Xcorr cutoffs of ≧1.8 for [M+2H]^2+^/2 ions and ≧2.5 for [M+3H]^3+^/3 ions. In addition, the database contained a concatenated reverse decoy database to estimate false-discovery rates, which were at 5% or below.

#### Immunofluorescence Microscopy

Immunofluorescence staining and microscopy procedures are described in Material S1.

## Supporting Information

Figure S1 Immunofluorescent analysis of p120 and cd98 in A431 and A431D cells.
(TIF)

Material S1 Supplementary methods (Immunofluorescence Microscopy), figure legend for figure S1, and legends for Tables S1 and S2.
(DOC)

Table S1 Background proteins detected in both p120 and negative control ReCLIP samples are shown.
(XLS)

Table S2 Complete peptide identification data (peptide sequence, cross correlation (X-corr) score, ion hits, and charge (z)) for proteins described in Figure 4.
(XLS)
