# Supplementary figures and images for: ReCLIP (Reversible Cross-Link Immuno-Precipitation): An Efficient Method for Interrogation of Labile Protein Complexes
Source: PLoS One. 2011 Jan 20;6(1):e16206. doi: 10.1371/journal.pone.0016206 (PMC3024417; doi:10.1371/journal.pone.0016206)

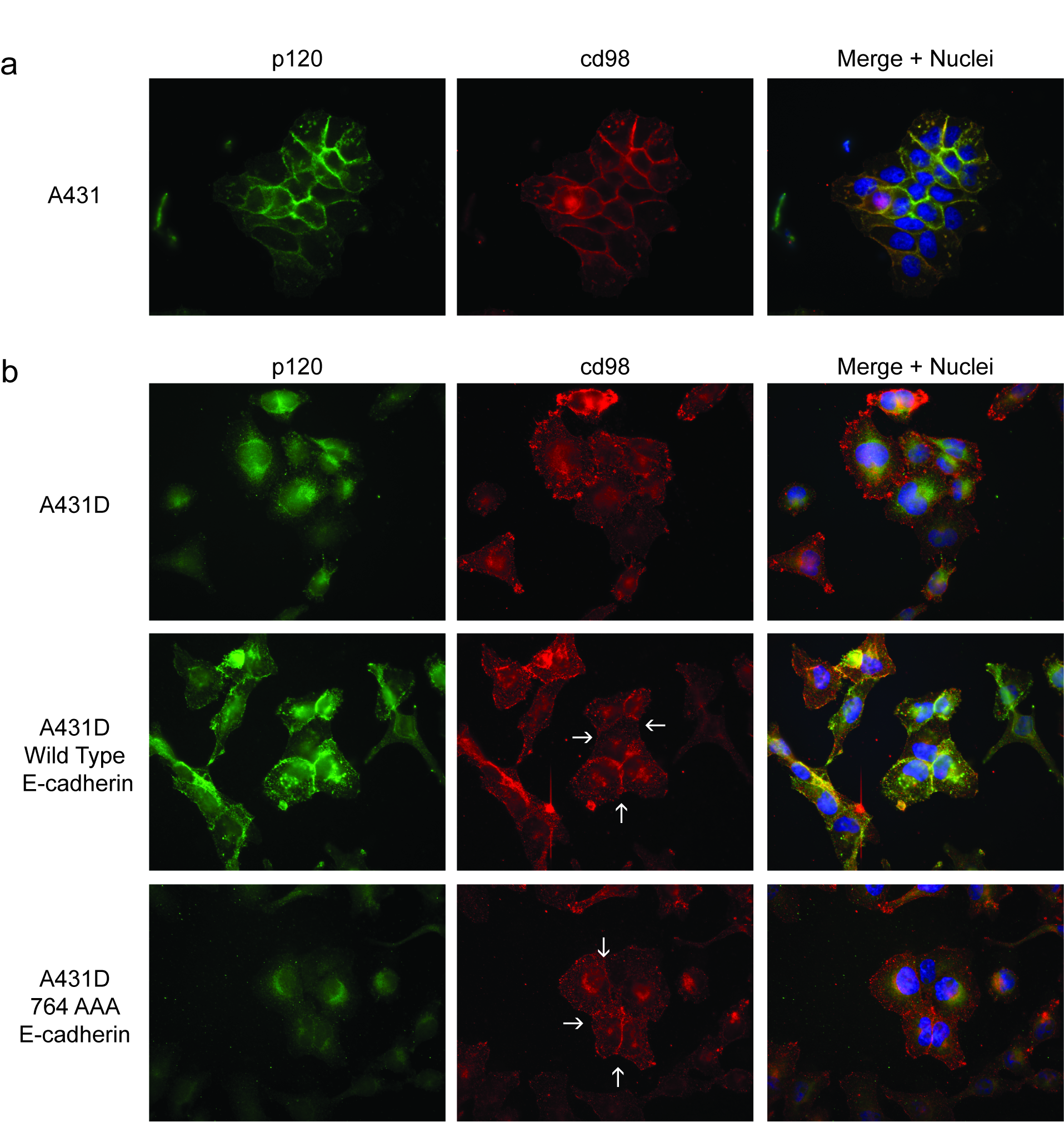

Supplement: Figure S1 — Immunofluorescent analysis of p120 and cd98 in A431 and A431D cells. (TIF) [file pone.0016206.s001.tif]
