# Supplementary material for: ReCLIP (Reversible Cross-Link Immuno-Precipitation): An Efficient Method for Interrogation of Labile Protein Complexes
Source: PLoS One. 2011 Jan 20;6(1):e16206. doi: 10.1371/journal.pone.0016206 (PMC3024417; doi:10.1371/journal.pone.0016206)
Supplement: Material S1 — Supplementary methods (Immunofluorescence Microscopy), figure legend for figure S1, and legends for Tables S1 and S2. (DOC) [file pone.0016206.s002.doc]

**Supplementary Methods**

**Immunofluorescence Microscopy**

A431 and A431-D cells were plated on glass coverslips 2 days before processing for immunofluorescence staining. Briefly, cells were fixed in 3% Paraformaldehyde for 30 minutes, and permeabilized in PBS/0.2% Triton X-100 for 5 minutes. Cells were blocked with PBS containing 5% BSA for 10 minutes. Cells were incubated with primary antibodies (anti-p120 pAb F1αSH, and anti-cd98 mAb 4F2) diluted in blocking buffer for 30 minutes, followed by secondary antibodies (anti-mouse IgG and anti-rabbit IgG conjugated to Alexafluor 488 or 594), for another 30 minutes. Cells were stained with 0.5 μg/mL Hoechst dye for one minute to stain nuclei. Coverslips with stained cells were mounted onto glass slides using Prolong Gold antifade reagent (Invitrogen) and imaged using a Zeiss Axiovert fluorescence microscope with a 63x objective. Images were acquired and processed using Metamorph software (Molecular Devices).

**Supplementary Figure Legends**

**Figure S1: Indirect association of p120 and cd98.** (a) Immunofluorescence analysis of endogenous p120 (green) and cd98 (red) in paraformaldehyde fixed A431 cells. Co-localization and nuclei (blue) is shown in the merged images. (b) Immunofluorescence analysis of endogenous p120 (green) and cd98 (red) in paraformaldehyde fixed parental A431D cells and A431D cells expressing wild type (Wild Type E-cadherin) or p120-uncoupled (764 AAA E-cadherin) E-cadherin. Arrows indicate junction-localized cd98.

**Supplementary Tables Legends (Tables are in separate excel documents)**

**Table S1: Background associated with ReCLIP.** Background Proteins detected in both p120 and negative control ReCLIP samples are shown with the highest single spectral count detected across all experiments. International Protein Index (IPI) Accession numbers are provided for each entry.

**Table S2:** Complete peptide identification data (IPI accession, peptide sequence, cross correlation (X-corr) score, ion hits, and charge (z)) for proteins described in Figure 4. Data is grouped according to each preparation analyzed by LC-MS/MS.
